# The assets-based approach: furthering a neoliberal agenda or rediscovering the old public health? A critical examination of practitioner discourses

**DOI:** 10.1080/09581596.2016.1249826

**Published:** 2016-10-25

**Authors:** Michael J. Roy

**Affiliations:** ^a^ Yunus Centre for Social Business and Health/Glasgow School for Business and Society, Glasgow Caledonian University, Glasgow, UK

**Keywords:** Assets-based approach, social enterprise, community health, critical realism

## Abstract

The ‘assets-based approach’ to health and well-being has, on the one hand, been presented as a potentially empowering means to address the social determinants of health while, on the other, been criticised for obscuring structural drivers of inequality and encouraging individualisation and marketisation; in essence, for being a tool of neoliberalism. This study looks at how this apparent contestation plays out in practice through a critical realist-inspired examination of practitioner discourses, specifically of those working within communities to address social vulnerabilities that we know impact upon health. The study finds that practitioners interact with the assets-based policy discourse in interesting ways. Rather than unwitting tools of neoliberalism, they considered their work to be about mitigating the worst effects of poverty and social vulnerability in ways that enhance collectivism and solidarity, concepts that neoliberalism arguably seeks to disrupt. Furthermore, rather than a different, innovative, way of working, they consider the assets-based approach to simply be a re-labelling of what they have been doing anyway, for as long as they can remember. So, for practitioners, rather than a ‘new’ approach to public health, the assets-based public health movement seems to be a return to recognising and appreciating the role of community within public health policy and practice; ideals that predate neoliberalism by quite some considerable time.

## Introduction

In the last couple of decades, there has been a sustained call to re-orientate public health more closely towards ‘enabling the growth of what nourishes human life and spirit, and supporting life’s own capacity for healing and health creation’ (Hanlon, Carlisle, Hannah, Reilly, & Lyon, 2011, p. 35). The so-called ‘assets-based approach’ to public health seems to be emblematic of this type of thinking: building upon the potential strengths of individuals and communities (Morgan, Davies, & Ziglio, 2010; Morgan & Ziglio, 2007) rather than focusing on individual risk factors such as smoking, alcohol, diet and exercise alone (Foot, 2012; Foot & Hopkins, 2010). Originally introduced into the field of community development as assets-based community development (ABCD), the argument goes that services often exploit need, disempower communities and produce clients. Communities are often defined in terms of problems (or deficits), with the result that individuals, families and even whole communities can be viewed negatively, either as definitive of certain issues, or even as problems in their own right (Brooks, Magnusson, Spencer, & Morgan, 2012; Evans & Pinnock, 2007). ABCD is considered as one method among many which aims to mobilise and harness the skills, resources and talents of individuals and communities. The central thrust is that communities should drive the development process themselves though identifying and mobilising existing – often unrecognised – assets and, in the process, respond to and create local economic opportunities. Kretzman and McKnight (1993) explain that assets include not only personal attributes and skills, but also relationships between people. Local associations and informal networks can lead to partnerships, which can leverage more formal institutional resources, such as from local government, third sector bodies and private (for-profit) enterprises.

With social determinants of health approaches finally reaching mainstream public health policy and practice in recent years, a clear need for new thinking has followed to shift emphasis away from viewing people as simply passive recipients of services and from ‘deficit-based’ policies and health promotion strategies (Brooks & Kendall, 2013). Public health policy-makers looking for new ideas to addressing the social determinants of health have thus found their way to the door of ABCD, with the ‘assets-based approach to public health’ (Morgan & Ziglio, 2007) the result.

However, ABCD – even when it was framed exclusively as a community development approach – has not been without its critics. These criticisms have inevitably been carried over as the approach has steadily and progressively started to influence public health policy rhetoric. Through analysing discourses of practitioners working in Scotland, where the assets-based approach has seemingly been enthusiastically embraced by both politicians and prominent officials alike (e.g. see Chief Medical Officer for Scotland, 2011), this paper seeks to critically examine the ways in which the assets-based approach has been understood, translated and then put into practice. In other words: to examine whether the existence and permeation of an ‘assets discourse’ through policy and into practice has made any difference to the way that community-based practitioners explain and/or consider their work in health terms. First of all, however: what is meant by public health assets? And what is the nature of the critique?

## On public health assets

Despite entering the lexicon of policy-makers, particularly over the last five years, the meaning of – and what constitutes – ‘assets’, at least in a public health context, has remained stubbornly conceptually imprecise. Brooks and Kendall (2013, p. 128), for example, explain that assets are ‘any factor that enhances the ability to create or sustain health and well-being, such as the resources that promote self-esteem and the coping abilities of individuals and communities’. Alvarez-Dardet, Morgan, Cantero, and Hernán (2015, p. 1), meanwhile, define public health assets as the:

expression of fair, equitable and democratic communities, resulting from their organized efforts; this is achieved by facilitating community empowerment and capacities which improves, promotes and restores the health of populations and can help to reduce health inequalities.

Glasgow Centre for Population Health profiled the work of 19 projects illustrating how asset-based approaches are applied in Scotland, including the work of a number of community-led organisations. Central to assets-based approaches, they say, is:

the idea of people in control of their lives through development of their capacities and capabilities. It is thought that such control enables people to become better connected with each other and encourages a spirit of cooperation, mutual support and caring. (McLean & McNeice, 2012, p. 6)

However, while certain elements of these definitions evoke social democratic principles, it is perhaps not altogether surprising that an approach stressing economic ideas and language (such as ‘assets’ and ‘deficits’), which focuses upon self-help and a lack of reliance upon outside agencies, particularly the state, has been the subject of fairly robust critique. Friedli (2013, p. 135), for instance, argues that ‘respect for people’s capacity for resistance (generally described as “resilience”) is abstracted from any analysis of social injustice or the causes of inequalities’ and so obscures the structural social forces and institutions that can create and perpetuate inequitable conditions. She argues that it is perhaps too easy to abstract psychosocial factors from the material realities of people’s lives as if they are unrelated to social and economic advantage, while leaving issues of power and privilege unchallenged. MacLeod and Emejulu (2014, p. 432) concur: they describe the rise of assets-based approaches in Scotland as ‘neoliberalism with a community face’ and ‘a capitulation to the rise of neoliberalism and its values of individualisation, marketisation and privatisation of public life’.

Burkett (2011a, p. 574), however, warns of the prospect of throwing the (assets) baby out with the (neoliberal) bathwater:

Certainly, these approaches throw up all the ideological tensions and polemics we now expect within neoliberal policy frameworks – the more benign communitarian discourses that replace a focus on social justice; the agenda of self-reliance that challenges more structural responses; and the individualism and competition that contrast with collective action as a basis for social change ... yet to reject asset based approaches for these reasons would be simplistic and overlook their radical possibilities.

Of relevance to the international context, a pragmatic vein is also apparent in Bull, Mittelmark, and Kanyeka’s (2013, p. 160) work in some of the world’s poorest communities. They warn that a focus on assets must co-exist with some measures of addressing social justice, but conclude that the assets-based approach to health and well-being is ‘not only appropriate, but … necessary’. Moreover, it is frequently acknowledged by those in leading public health roles (see e.g. Tannahill, 2012) that adopting an assets-based approach, is not – and should not be seen as – a replacement to addressing the social determinants of health. It is therefore not controversial (at least in Scotland) to state that ‘community assets can only have a mitigating effect on the structural and social determinants of ill-health and inequality - poor housing, low wages, lack of jobs’ (Foot & Hopkins, 2010, p. 12).

So while it is clear that the intentions, meanings and political underpinnings of assets-based public health are contested – at least in academic discourse – it is not clear how, or whether, this contestation is played out at the level of public health practice. This paper attempts to address this gap by investigating the perspectives of practitioners, in cognisance of ‘the perils of invoking neoliberalism in public health critique’ (Bell & Green, 2016). Given the centrality of community to the assets-based approach, it was considered crucial to gain the perspectives of those who work in communities, rather than in formal health systems, to address particular social vulnerabilities that we know impact upon health. Research on the important role of organisations such as community-led social enterprises, which lie outside of formal health systems but impact on health and well-being through working to mitigate or alleviate local social vulnerabilities (see e.g. Farmer et al., 2016; Roy, Donaldson, Baker, & Kay, 2013; Roy, Donaldson, Baker, & Kerr, 2014), including in relation to neoliberalism (Roy & Hackett, 2016) is steadily increasing in prominence.

As both Tenbensel (2006) and Wesselink, Colebatch, and Pearce (2014, p. 342) have recognised, the need to be able to ‘read’ context, and ‘mobilise the appropriate discourse’ is well known to practitioners, and so a critical study of discourses employed by social enterprise practitioners was undertaken, with a view to going beyond surface impressions to explore whether the ‘assets-based public health’ movement is *actually* informing practice, and/or whether it is being resisted.

## Methodology and methods

The study was undertaken in Glasgow, a city in west-central Scotland, which has some of the poorest health in western Europe and a health profile more in common with eastern European countries (McCartney, Collins, Walsh, & Batty, 2012). Attempting to tackle the significant problem of health inequalities in Scotland has been a central policy agenda of successive Scottish Governments. The idea that there are untapped strengths within Scotland’s communities, many of which had suffered significantly over many decades through deindustrialisation and disinvestment, was a sufficiently powerful message to capture the imagination of influential (and often medically trained) actors within health policy circles seeking ‘alternative ways of working’ (Chief Medical Officer for Scotland, 2011, p. 26) to address the social determinants of health. Their influence precipitated significant policy traction for the assets-based approach. Moreover, Glasgow has a well-developed social enterprise sector; a recent report (GSEN & Social Value Lab, 2013) describes social enterprise in Glasgow as having ‘scale as well as substance’ and estimates some 509 social enterprises operating within the city, with a combined turnover of £767 m and employing over 10,000 people.

Where the research aim is to move beyond surface appearances to explore the processes involved, it is appropriate to study individuals in context (Sayer, 2000) and so in-depth, one-to-one semi-structured interviews were undertaken with social enterprise practitioners, and a focus group. It should be noted that an investigation of the assets-based approach was not the principal aim of the study from the outset; rather this was to investigate how social enterprise practitioners conceptualise their impact upon the health and well-being of the people they support, regardless of whether they explicitly *intend* to do so or not. A purposive, maximum variation sample (Mason, 1996) of social enterprises based in the city was therefore identified in order to make sense of the heterogeneity of experiences and practices of different social enterprises and contexts in Glasgow.

Practitioners were deliberately sought out who had both sufficient operational knowledge and day-to-day familiarity with the people they support. In smaller organisations, this was often the leader, but in larger organisations the most appropriate person was often found several rungs down the management ladder. They were identified and recruited by combining a data-set of social enterprises provided by a Glasgow-based enterprise support agency with contacts and personal knowledge of the sector. Seven of the social enterprises stated that impacting upon health and/or well-being was a key aim, while the remaining six did not explicitly mention ‘health’ and/or ‘well-being’ in their social mission, which was established from reading publicly available company documents. Data from these documents were used to support the process of developing key criteria in order to construct the sample. The sample of social enterprises was then constructed based upon maximum variation on these criteria, including: the industrial sector in which they operate; their geographical reach; turnover; length of time in operation; and number of employees. Overall, data were gathered from 13 different social enterprises between October 2013 and February 2015. The name of each organisation was anonymised and the name of the practitioner was disguised in alignment with the code name given to the social enterprise, so the name of the social enterprise practitioner based at Alpha was called Alan (a male respondent), while Christine (a female practitioner) was based at the social enterprise assigned the code Charlie. Ethical clearance for the study was obtained from Glasgow Caledonian University prior to data collection, and informed consent was sought and provided by every interviewee. Whether data saturation ever truly occurs is a long-standing debate, but the principle of generating *enough* data to inform the theory-building process was followed. Guided by the critical realist philosophical framework, (Bhaskar, 1975, 1987) an ‘abductive’ (Peirce, 1932; Timmermans & Tavory, 2012) approach to analysis was employed, which involved ‘moving backward and forward among empirical data, research literature, and emergent theory’ (Dey & Teasdale, 2013, p. 255) in an iterative manner to gain insights into the underlying structures and mechanisms that account for the phenomena involved.

The assets-based approach was raised spontaneously by several practitioners within the in-depth interviews, and so additional interviews and a focus group were undertaken to investigate the topic in much greater detail (making 15 interviews and a focus group in total). All of the interviews and the focus group were recorded and transcribed ‘intelligent verbatim’. Once satisfied that each transcript was an accurate representation of the interview, all of the data were imported into the computer-assisted qualitative data analysis programme QSR NVivo and a method of analysis and coding inspired by the critical realist philosophy, causation coding (Saldaña, 2013), was employed.

## Findings

In order to aid understanding, the findings are organised into three discrete sections. First of all, *how* the practitioners conceptualised their impact upon health and well-being is explained. Secondly, an explanation of *why* they conceptualised their impact in such ways was explored. Thirdly, critical reflections on whether the assets-based public health influenced the way that they explained their impact is explored in greater depth, with particular focus on whether the assets-based approach was considered novel to the way that they approached or explained their work.

### Impact upon health

Many of the social enterprises examined operate in communities that are (perhaps by ‘professional’ definition) ‘asset poor’; that is, suffering from significant material disadvantage. It is therefore not surprising that several of the social enterprises engaged in activities that, it could be argued, were decidedly ‘deficits based’: working to help improve literacy skills and knowledge, for example, with a view to improving someone’s employability prospects. Although the social enterprises could be seen to be focusing upon complex issues related to various aspects of social vulnerability, several of the social enterprises cautioned against defining communities in purely negative terms, and considered that it was helpful to recognise the presence of various forms of assets which social enterprises can build upon and mobilise for the benefit of communities, not least to encourage self-reliance.

The topic of assets was brought up spontaneously by Jill during the focus group. She was familiar with the assets-based literature, after initially training as a community development worker, and cautioned that communities should not be defined simply in terms of vulnerabilities or deficits:

It’s about capitalising upon the assets of communities as well, so it isn’t just about vulnerabilities, it’s about the capacity of communities. (Jill)

Jill then drew upon an example of being able to draw on a pool of willing volunteers with a blend of experiences and strengths. This provoked Martin and Laurence, also in the context of the focus group, to mention the use of space and common areas for environmental improvement for the social and economic development of their respective communities.

When asked: ‘some people have said that social enterprises, through doing such work, can improve people’s health. What do you think about that?’ it was apparent that almost all of the social enterprises exhibited a highly nuanced and sophisticated understanding of the impact they were having. While not all of them expressed their impact in health terms before that point, every one of the practitioners considered that working to improve social connectedness and social cohesion was key to improving people’s lives, and most of them then extended this rationale to health after they were asked. For example, Bill’s reply to that question was unequivocal:

That’s why I’ve done it! That’s why we do it! Half of the bloody stuff that kicks about in this notion about integration is people’s fears and the propaganda that plays on people’s fears. People get scared of the unknown. You see somebody from a different culture who talks a different language who dresses different, oh there’s a scary person, for whatever reason. When you generate fear in people, it’s not healthy to be living in constant fear of stuff like that.

So the idea behind the whole international festivals and the carnival and all of that that we had, was about trying to celebrate cultural diversity by bringing people out and engaging with people … It was about breaking down those fears and unknown barriers. Definitely that sort of stuff helps in health and well-being. People feel connected, they feel part of something, they take a sense of pride, it builds confidence in people. (Bill)

Surprisingly, only one of the practitioners provided an answer to that question in such a way that suggested that a rather narrow – medical – conceptualisation of health:

we provide young people with water, fruit, fruit juice, healthy snacks and we try and encourage that because a lot of the young people that we work with live off energy drinks, they smoke … it takes a while for them to understand that because they are eating really badly that is why they don’t have the energy and they are having the highs and the lows. (Fiona)

It could reasonably be inferred, because of her reference to health ‘risk factors’ such as smoking and diet, that Fiona did not fully appreciate the link between her own day-to-day work in relation to improvements in confidence and the progression of the young people she supports, with health and well-being. These aspects had, in her estimation, intrinsic value in and of themselves. However, it does raise an interesting question as to why more of the practitioners did not also think along such lines. Given that the assets-based approach to public health is built upon, and advocates for, a holistic definition of health, perhaps this indicated that the assets-based policy message had taken hold? The data were thus revisited to explore this question in greater depth.

### How are assets understood?

When she was asked to explain her understanding of what she thought the assets-based approach meant, Polly explained that community-based organisations not only acted as assets in their own right, but also as mechanisms to build and maintain other forms of asset, and the networks between them, and between people:

‘I like to think of it quite simply … just using the assets that are within a community to improve people’s health and well-being. I don’t think it needs to be more overly complicated than that.

[Assets are] people really, and networks. I guess any kind of community based organisation. Really for me it is about people building those networks and ensuring that our older people are connected with services and our young people are connected with services … I think that if you have got good social networks and people are well connected then it is connected to public health.’ (Polly)

Jill, on the other hand, described the assets-based approach in a more nuanced and holistic way, stressing the importance of networks, but also in relation to meaning in people’s lives (which she describes as ‘what matters to people’):

The approach involves working with people and the community, and in where they live in their locality and their local neighbourhood, to look at *what matters to them*, not *what is the matter with them*. What matters to them and how they can achieve the best that they can achieve in their circumstances, whether that is to be less isolated, to be healthier, to eat healthier, to look after their children in a healthier way, and use what they have, where they start from, how they use their knowledge, their skills, their abilities, their links to do something about it. Social enterprises are facilitators in that: the catalysts. We’re not there to do it, or to tell, or to design; we are there to unlock it, to facilitate and to maybe give little prompts, you know ‘try this, try that,’ and be able to facilitate it. (Jill)

According to Jill, the role of her organisation is therefore to act as a catalyst or facilitator within a local community, to work out what matters to them, rather than focus upon the deficit: what the matter is. According to her, it is not her job to ‘fix’ people or problems, in the manner of a doctor or an engineer, but to support people to fulfil their potential to live more fulfilling and happy lives. Such an approach is consistent with what Whitehead (2007, p. 474) recognises as building the resilience of individuals in disadvantaged circumstances through ‘person-based strategies’, part of a much broader series of actions aimed at reducing health inequalities through:

moving away from deficit models, towards recognition of the assets and capabilities that individuals with adversity possess. The logic in this case is that interventions that acknowledged these positive strengths and removed barriers to their realisation would release capacity to act in ways that improved health and quality of life among the worst-off in society.

### Continuity with the past?

Polly insisted that the practices of social enterprise practitioners were consistent with the assets-based approach, but this was not a recent development:

A lot of the time it is like, ‘But we are doing this anyway, why do I have to use that language?’ … I think just in terms of the whole asset based approach I think that it is something that is right at the heart of what social enterprise is. (Polly)

The idea of continuity with the past was also an idea raised by Jill:

We’ve been doing this long before my time, they’ve been doing it in this building for 30 years but it’s called something different, community action maybe then community development, community education, whatever; and now it’s community assets whatever it will be in the next decade, I don’t know, but it’s about the values that underpin that and it’s about people and communities helping each other help themselves. (Jill)

Jill explained that such work has been known by different names at various times, likely reflecting different ‘policy cycles’ (Howlett, Ramesh, & Perl, 2009) but essentially related closely to the consistent thread of the approach taken by her organisation and a commitment to a set of values related to ‘encouraging and nurturing people’ over many years.

## Discussion

Like the assets-based approach, social enterprise has also been accused of being an ‘embodiment of a neoliberal welfare logic’ (Garrow & Hasenfeld, 2014). In his analysis of social enterprise discourses, Teasdale (2012, p. 107) shows how social enterprise has been presented by critics as ‘one element of a neoliberal grand narrative’. However, similar to Burkett’s (2011a, 2011b) argument earlier in response to critics of the assets-based approach, Gibson-Graham (2008, p. 618) argue that experimental forays into building new economies, such as upon social enterprise, are often dismissed as capitalism in another guise, already co-opted and/or judged to be inadequate before they are explored ‘in all their complexity and incoherence’. Such arguments parallel the discussion in relation to the assets-based approach to public health, and the question of whether or not the practitioners studied are simply ‘unwitting tools’ of neoliberalism. However, rather than simply being yet ‘another reflection of neoliberal hegemony’ (Bell & Green, 2016, p. 241) it was clear, at least to the practitioners in question, that their intention was to help those most at risk in society from, and most affected by, the forces that we know to be responsible for health inequalities, such as the ‘toxic combination of poor social policies and programmes, unfair economics, and bad politics’ (Commission on Social Determinants of Health, 2008, p. 35).

In terms of the impact that social enterprises were having upon health, it was found that the Scottish Government’s ‘assets-based agenda’ was explicitly or implicitly echoed in the discourses of many of the social enterprise practitioners. They presented a sophisticated explanation of the impact their work was having; consistent, in fact, with a holistic and progressive conceptualisation of health and well-being. Although this was initially thought to be a by-product of the assets policy discourse permeating down and influencing their work, a different explanation was found: that many of them were, deliberately or otherwise, simply following what they considered to be good community development practice. It was also considered whether practitioners were employing tactics of ‘everyday’ micro-resistance (viz. de Certeau, 1984) such as through reproducing or mimicking the policy discourse, a behaviour that Dey and Teasdale (2016) coined ‘tactical mimicry’ to mean that they were acting as if they were simply falling in with the prevailing policy discourse, perhaps for reasons of securing legitimacy and/or resources. Such an idea becomes conceivable if the proximity between social enterprises and the public sector in Scotland and Glasgow is considered, the seeming ubiquity of assets-based language and ideas across many areas of social policy in Scotland, and the power of the message itself. On this latter point, Friedli (2013, p. 131) observes that ‘the emergence of asset-based approaches to improving health is generating a level of evangelism not seen since the days when social capital, a not unrelated construct, inspired a similar fervour’. However, the explanation from this research was found to be quite the opposite: the ideas were not being reproduced, or indeed, resisted at all. Rather, the policy direction and discourse was instead found to have caught up with social enterprise practice. The social enterprise practitioners studied were found to be very aware of the material realities of the people they work to support; indeed, such first-hand knowledge is one of their principal strengths. So rather than ‘participating in an individualisation agenda’, they were rather more interested in working to mitigate the worst effects of poverty and social vulnerability at the local level, particularly through enhancing collectivism and solidarity, bringing people together to address social cohesion in communities and recognising – often instinctively – that social ties are critical to well-being. These are all facets that neoliberalism arguably seeks to disrupt.

Furthermore, rather than being novel, ‘assets-based ideas’ were simply seen as the latest iteration of a continuing focus on empowering people, and supporting people to empower themselves, ideas that are commonplace within the social enterprise and social economy movement and (to their recollection) always had been. The ideas presented as being ‘new’ in public health policy discourse, have, in fact, been employed for many years – possibly even many decades – by organisations such as community-based social enterprises and predecessor forms of organisation that have been around in Scotland for some considerable time. This notion of continuity of an old idea finds its parallels with recent attempts to redirect the attention of public health theorists and practitioners back towards structural and environmental influences on health and health behaviours. Rather than a new way of working, this is, as Macintyre, Ellaway, and Cummins (2002, p. 127) point out, ‘actually a reorientation back to what some would regard as “the old public health”, that is, the nineteenth and early twentieth century attempts to clean up dirty cities, and to move away from the late twentieth century concern with chronic disease and contributory individual lifestyles’. This is an idea also raised by Lomas (1998, p. 1187) who also recognised that re-balancing public health – complementing individualistic biomedical and economic views of the world with a social science focus on community and societal structures – is not a new idea, but actually a return to those of early nineteenth-century pioneers of public health and epidemiology.

## Conclusion

This study set out to critically examine the ways in which the ‘assets-based approach’ to public health has influenced the work of practitioners; whether it was recognised as a potentially empowering means for communities to address the social determinants of health, or, in contrast, simply furthering a neoliberal agenda. It was found that practitioners interact with the assets-based policy discourse in interesting ways: rather than unwitting tools of neoliberalism, they considered their work to be about supporting communities to work together to mitigate the worst effects of poverty and social vulnerability at the local level.

Framing the assets-based approach as simply furthering a neoliberal agenda downplays, or perhaps fails to appropriately consider, the agency of practitioners in resisting, (de-) constructing and utilising policy ideas and discourses – even those that could reasonably be argued as being ostensibly neoliberal in nature – to suit their own agenda; to benefit the individuals and communities they exist to support.

Rather than a different, innovative, way of working, practitioners considered the assets-based approach to simply be a re-labelling of what they have been doing anyway. So rather than a ‘new’ approach to public health, the assets-based movement seems to be indicative of a return to recognising and appreciating the role of community within public health policy and practice.

There are, of course, a number of limitations of the study. The specific location and context for the study potentially limits the transferability of the findings. Moreover, it is not inconceivable that the practitioners studied may have over-accentuated the positive impacts of their work, given that they are often called upon to justify their existence to a range of stakeholders including their boards, communities, and, most commonly of all, funders. In the future, it would be useful to explore not only how practitioners *explain* their work, but the everyday practice of ‘doing’ social enterprise. Ethnographic methods may be useful to explore this dimension.

The findings have clear policy implications. It is considered that both agency *and* structure need to be sufficiently taken account of when seeking to address the social determinants of health. By operating outside of formal public health systems, social enterprise practitioners would not have been influenced by the ‘methodological and theoretical individualism’ (Macintyre et al., 2002, p. 126) that can often characterise modern mainstream public health research and practice. Not only is the knowledge of ‘lay’ people working outside of formal public health systems crucial to addressing long-standing issues of public health concern (Popay & Williams, 1996; Popay, Williams, Thomas, & Gatrell, 1998), but also that the public health impact of the work of community practitioners operating outside of formal health systems requires to be better appreciated and understood. Finally, the findings re-emphasise that message that processes of policy-making and subsequent implementation are in no way straightforward or inevitable.

## Disclosure statement

No potential conflict of interest was reported by the author.

## Funding

This work was supported by the Economic and Social Research Council and Medical Research Council [grant number MR/L0032827/1].
